# UV-Induced Photocatalytic Cashmere Fibers

**DOI:** 10.3390/ma10121414

**Published:** 2017-12-11

**Authors:** Lingyun Wang, Walid A. Daoud

**Affiliations:** School of Energy and Environment, City University of Hong Kong, Tat Chee Avenue, Kowloon, Hong Kong, China; lingywang8-c@my.cityu.edu.hk

**Keywords:** anatase, cashmere, photocatalysis, superhydrophobicity

## Abstract

Cashmere with UV-induced photocatalytic properties is developed for the first time by applying nanocrystalline anatase TiO_2_ colloid that is free of inorganic acids and organic solvents via a facile low-temperature one-step sol-gel process. The coated cashmere exhibits remarkable UV-induced photodegradation of methyl orange. Furthermore, the photocatalytic nano-coating on cashmere exhibits significant stability after repetitive washing cycles without the need for chemical or physical pretreatment, where the photocatalytic activities remain almost unchanged after three washing cycles while maintaining a water contact angle above 150°. The one-step functionalization process also minimizes the impact on the peculiar intrinsic properties of cashmere. These findings indicate that cashmere combining reproducible UV-induced photocatalytic activity with stable superhydrophobicity has potential in practical applications.

## 1. Introduction

Self-cleaning materials have received considerable attention due to their unique properties and practical applications in the field of energy and environment, such as solar panels, windows, fabrics, and paints [1,2,3]. Photocatalytic textiles, with the properties of self-cleaning, anti-pollution, deodorization, and antimicrobial effects, are particularly of great interest in terms of sustainability [4]. In general, there are two categories of self-cleaning surfaces. One is superhydrophobic surfaces, commonly known as the “Lotus effect,” with a water contact angle (WCA) larger than 150°. When water droplets promptly roll off the surface, contaminants are consequently removed. Many researchers have developed superhydrophobic coatings on surfaces by solution-dipping [5,6], sol-gel processes [7,8], one-pot hydrothermal reactions [9], and plasma treatments [10], with the aim of constructing hierarchical micro/nano structures to increase surface roughness and modifying the surface energy, which are the two significant factors affecting surface wettability.

The other is superhydrophilic surfaces, with a WCA smaller than 10°. Self-cleaning hydrophilic surfaces mainly involve a photocatalysis process, where stains and dirt on the surface are broken down into CO_2_ and water under the exposure of UV or visible light. Nano-crystalline anatase TiO_2_ has proved to be one of the most investigated photocatalyst, owing to its unique physical and chemical properties, such as its efficient photocatalytic activities, high stability, nontoxicity, and cost-effectiveness [11,12]. A surface that possesses both photocatalytic and superhydrophobic properties could form the ideal self-cleaning surface for practical applications. In this regard, attempts have been made to develop textile surfaces with photocatalytic and superhydrophobic properties. A TiO_2_-SiO_2_@PDMS hybrid film was prepared via a sol-gel process for polyester-cotton fabrics, with wash and acid resistance as well as the advantage of photocatalytic property of TiO_2_ [7]. In addition, a superhydrophobic cotton textile with a WCA of 156° and superior photocatalytic activity under visible-light was developed through anatase TiO_2_ coating in conjunction with meso-tetra(4-carboxyphenyl) porphyrin, followed by modification with trimethoxy(octadecyl)silane [13]. Conventionally, it is a two-step process for a surface to acquire both superhydrophobic and photocatalytic properties. Furthermore, these functional textiles are cellulosic, have the capability of enduring harsh experimental conditions, and can undergo multi-step processing. Here, we investigate cashmere, a keratinous protein fiber with unique properties, such as softness, insulation, durability, and warmth, which is renowned for being delicate and difficult to care for in comparison with other fibers. As such, it has been more challenging to modify cashmere because of its inferior chemical and thermal resistance, photostability, and resistance to degradation [14]. Therefore, it is essentially important for cashmere to acquire self-cleaning properties using a facile and compatible process without impairing its intrinsic properties. Although there have been a few studies carried out on the functionalization of wool, which is also a keratinous protein fiber [15,16,17,18,19,20,21], additional chemical or physical treatments, such as surface succinylation [15] or plasma treatment [20], were needed to confer a photocatalytic self-cleaning property. Subsequently, the enrichment of carboxylic groups of wool surfaces allowed the enhancement of bonding between wool and photocatalyst. In contrast, it was found here that, without prior chemical or physical modification, the nano-coating cashmere fabric exhibits good bonding behavior, indicating stability after repetitive washings. 

Herein, in this contribution, we developed photocatalytic self-cleaning cashmere by adopting a facile one-step process without additional treatment. In view of its characteristics and hydrophobic nature, a low temperature sol-gel process using a devised TiO_2_ colloid was developed. The conventional synthesis of TiO_2_ colloid involves inorganic acids, such as hydrochloric acid [22,23,24] and nitric acid [25,26], as well as organic solvents [17]. Here, the TiO_2_ colloid was prepared without the use of inorganic acids or organic solvents to minimize the impact on cashmere fibers. To our best knowledge, this is the first time cashmere has been endowed with a photocatalytic self-cleaning property. The TiO_2_-coated cashmere fabric not only exhibited UV-induced photocatalytic and superhydrophobic properties but also showed remarkable wash stability. 

## 2. Materials and Methods

### 2.1. Synthesis of TiO_2_ Colloid

TiO_2_ colloids were prepared using a devised method. Titanium tetraisopropoxide (TTIP, 97%, Sigma Aldrich, St. Louis, MO, USA) with concentrations of 5% and 10% of total volume amount was added to a mixture of glacial acetic acid (99.8%, International Laboratory, South San Francisco, CA, USA) and deionized water (18.2 MΩ·cm). The mixture was heated at 60 °C under vigorous stirring for 2 h.

### 2.2. Preparation of TiO_2_-Coated Cashmere Fabrics

Cashmere fabric was first cleaned with a nonionic detergent (Kieralon F-OLB, Shanghai, China, BASF, Ludwigshafen, Germany) at 40 °C for 30 min to remove all the impurities. The TiO_2_ colloid was then applied to the cleaned cashmere fabric (15 × 15 cm^2^) through a dip-pad-dry-cure process [27]. The scoured cashmere fabric was dipped into the TiO_2_ colloid at room temperature and then pressed with a pneumatic press (MU505C, FYI, Hefei, China) at a pressure of 0.4 MPa and a roller rotation speed of 10.5 rpm. Afterwards, the sample was dried at 60 °C in a drying oven for 5 min and cured at 120 °C for 3 min. Finally, the fabric was washed under water flow until the surface pH reached 7 and air dried.

### 2.3. Characterization

The crystallinity of the solid powder extracted from the TiO_2_ formulations was studied by X-ray diffraction spectroscopy (XRD). The XRD spectra were recorded on a Rigaku Smartlab X-ray diffractometer with Cu Kα radiation (λ = 1.5406 Å) operating at 200 mA and 45 kV in the region of 2*θ* = 20°–70°. The grain size of TiO_2_ nanoparticles was calculated according to the Scherrer equation *D* = (*K λ*)/(*β cos θ*), where *D* is the crystallite size, *K* is the shape factor 0.9, *λ* is the wavelength, *β* is the full width at half maximum (FWHM) of the corresponding peak, and *θ* is the Bragg angle. The crystallinity of TiO_2_ coating on cashmere fabric was also determined by XRD. The morphology of solid powder extracted from the 5% and 10% TiO_2_ colloids was observed with transmission electron microscopy (TEM, JEOL JEM-2100F, Tokyo, Japan) operated at 200 kV. The surface morphology of the cashmere samples was characterized using field emission scanning electron microscopy (FESEM, JEOL JSM-6335F, Tokyo, Japan) at an accelerating voltage of 5.0 kV. The UV-vis absorption spectra of pristine and coated cashmere fabric were recorded on a UV-vis spectrophotometer (UV-2600, Shimadzu, Kyoto, Japan) using a 60 mm integrating sphere with BaSO_4_ as reference. Water contact angle (WCA) of the cashmere fabric was recorded on an optical contact angle system (Krüss, Hamburg, Germany) using 5 µL of deionized water droplet at room temperature.

### 2.4. Photocatalytic Studies

Photocatalytic degradation of methyl orange (MO, International Laboratory, South San Francisco, CA, USA) was evaluated quantitatively to assess the self-cleaning properties of the nano-coating on cashmere fabric. Coated and pristine cashmere specimen (1.5 × 1.5 cm^2^) were immersed in crystallization dishes containing an MO solution (20 mL, 15.3 µM). The dishes were placed on a bench-top shaker inside a light box equipped with a ventilation exhaust fan for maintaining the ambient temperature and irradiated with UV light (130 µW/cm^2^, wavelength centered at 365 nm) for 3 h. Prior to UV irradiation, the samples were kept in the dark for 30 min to attain adsorption-desorption equilibrium. At given time intervals, 2 mL of solutions were sampled and analyzed using UV-vis spectroscopy, and the change of MO concentration was monitored at a wavelength of 464 nm. 

### 2.5. Stability Study

The wash stability of TiO_2_ nano-coating on cashmere fabric was tested against nonionic detergent (Kieralon F-OLB, BASF) using a modified AATCC Test Method 190–2003 [28]. Samples were washed with the detergent solution (2 g/L) for 45 min at room temperature at a constant stirring of 400 rpm. The samples were then washed with water and air dried. The washing test was repeated three times to assess the stability of the nano-coating after washing. The photocatalytic activity of the cashmere fabric after each wash cycle was tested through MO degradation.

## 3. Results and Discussion

**Figure 1:**
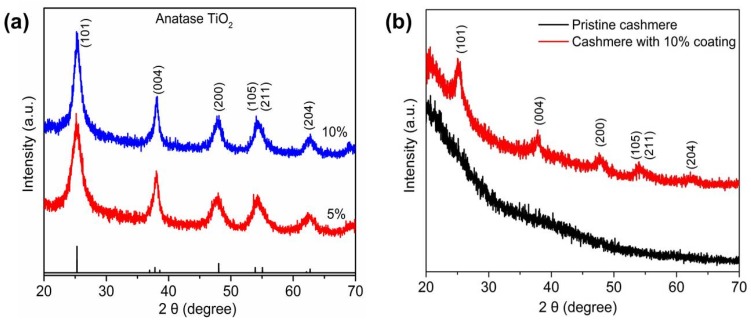
XRD spectra of (**a**) solid extracts from 5% and 10% TiO_2_ colloids, (**b**) cashmere fabric with 10% coating and pristine cashmere fabric.

Figure 1 shows the XRD spectra of solid extract and coated cashmere fabric. In Figure 1a, the peaks at 2θ values of 25.3, 37.8, 48.0, 54.0, 55.1, and 62.7 can be indexed to (101), (004), (200), (105), (211), and (204) planes of anatase TiO_2_, respectively. The XRD spectra demonstrate that the precursor concentration does not impact on the lattice structure of TiO_2_. Calculated using the Scherrer equation and the (101) plane, the crystallite size of 5% and 10% TiO_2_ was 7 and 9.6 nm, respectively. In addition, the crystallinity of TiO_2_-coated cashmere fabric was studied by XRD as shown in Figure 1b. In order to attain high signal-to-noise diffraction peaks, 10% TiO_2_ with four coatings were applied to cashmere fabric. Compared to pristine cashmere, coated fabric displayed the characteristic peaks of anatase TiO_2_. Furthermore, the calculated crystallite size of 9.7 nm is in good agreement with that of the 10% solid extract.

**Figure 2:**
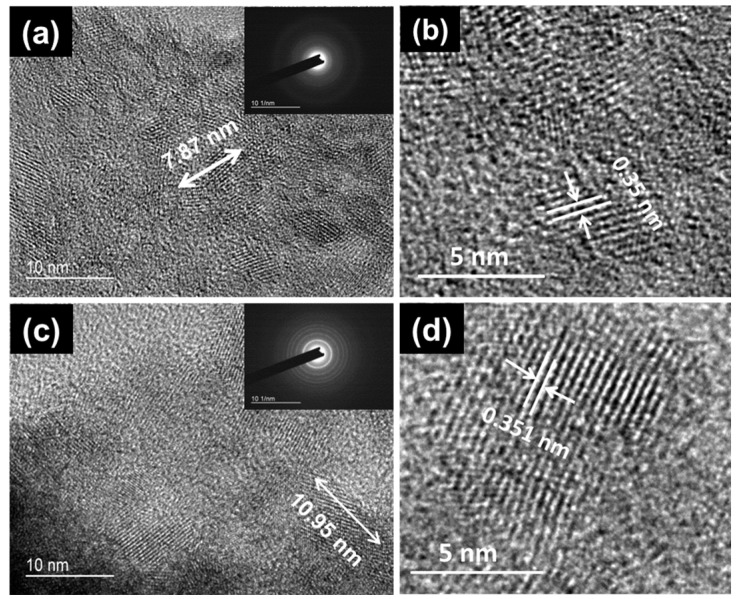
HRTEM images of (**a**,**b**) 5% TiO_2_ and (**c**,**d**) 10% TiO_2_. Insets in (**a**,**c**) show the corresponding SAED.

TiO_2_ extract from the 5% and 10% formulations was further investigated by HRTEM. Figure 2a,c shows that the nanoparticles have a needle-like shape. The congregated nanoparticles reveal that the interaction between the nanoparticles was strong enough to bear the ultrasonication during the sample preparation for TEM analysis, which is consistent with Wong et al. [29]. The grain size of the 5% and 10% TiO_2_ were 7.87 and 10.95 nm, respectively, matching well with the size derived from the XRD analysis. Furthermore, Figure 2b,d indicates high crystallinity with the lattice spacing of 0.35 and 0.351 nm for 5% and 10% TiO_2_, respectively, which agrees well with the interplanar spacing of the (101) plane of anatase TiO_2_. Both the selective area electron diffraction (SAED) patterns (insets in Figure 2a,c) further confirmed the TiO_2_ crystals were of anatase form.

**Figure 3:**
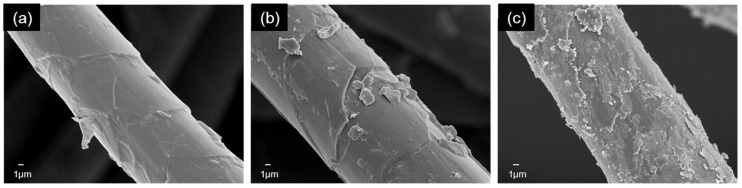
SEM images of cashmere fabric (**a**) pristine (**b**) with 5% coating and (**c**) with 10% coating.

The surface morphology of pristine and coated cashmere is illustrated by FESEM images in Figure 3. It is noted that the scales are fewer and much thinner in cashmere, which is significantly different from our previous study on wool [14]. In addition, a clear difference in surface morphology can be seen when comparing cashmere fibers before and after coating. For 5% coated fiber (Figure 3b), except for some aggregations outside the fiber, which may be ascribed to either the congregate of TiO_2_ nanoparticles or the cashmere fiber, the surface is relatively even compared with 10% coated fiber. Due to the higher concentration and coating process, the cashmere fabric with a 10% coating (Figure 3c) appeared to have a rougher surface than the 5% coating (Figure 3b), displaying a coarse and uniform distribution of TiO_2_ on the fiber. The corresponding EDX of the 10% coated cashmere fabric (Figure S1) clearly shows the presence of TiO_2_ on the fiber. In comparison with the atomic ratio of other elements (C, O, and S) on cashmere, the TiO_2_ coating on cashmere fiber is relatively low.

The UV-vis diffuse reflectance spectra of pristine cashmere and TiO_2_-coated cashmere fabric are displayed in Figure S2. It can be noted that 5% and 10% TiO_2_-coated cashmere exhibited the same diffuse reflectance spectrum and the nano-coating had little influence on the optical properties of cashmere. Compared to pristine cashmere, the slightly decreased reflectance in 300–387 nm of the coated one is attributed to the increased UV absorbance of anatase TiO_2_. Meanwhile, the lower visible light absorption ability of TiO_2_ accounts for the enhanced reflectance in the visible region after coating.

**Figure 4:**
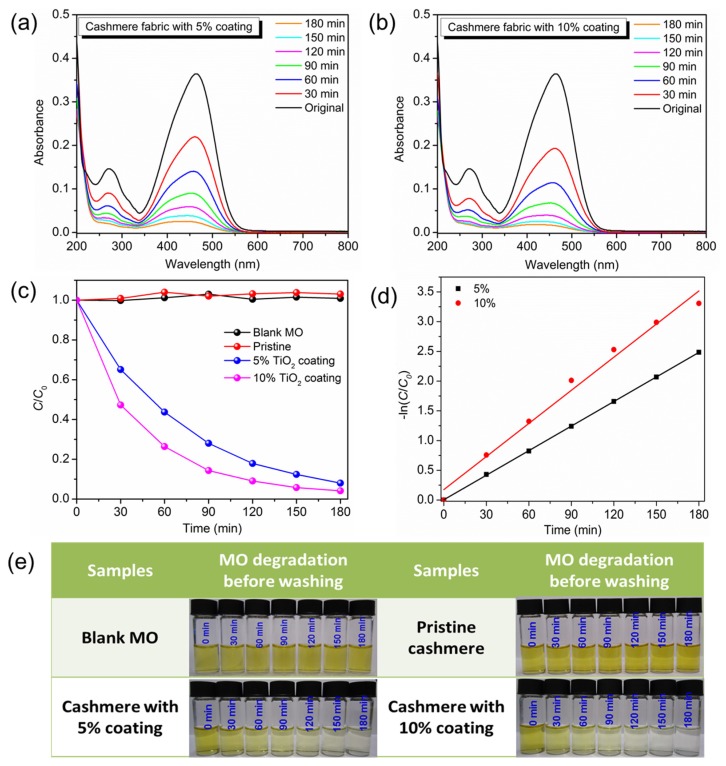
(**a**,**b**) Absorption spectra of MO in the absence and presence of cashmere fabric (pristine, 5% and 10% TiO_2_ coating). (**c**) Photocatalytic activities of blank MO, pristine cashmere, and cashmere fabric with 5% and 10% TiO_2_ coating. (**d**) Pseudo-first-order kinetics curves of MO degradation by coated cashmere fabric. (**e**) Photos of the MO solution in the absence and presence of the cashmere samples.

Cashmere fabrics with TiO_2_ nano-coatings were subjected to quantitative analysis through photocatalytic degradation of MO under UV light irradiation. Figure 4a,b shows the absorption spectra of MO in the absence and presence of cashmere with 5% and 10% TiO_2_ nano-coatings. The absorption peaks of all samples decreased with the increase in degradation time. Figure 4c shows a plot of normalized concentration of MO (C/C_0_) against time. For comparison, a blank MO solution and pristine cashmere fabric were also studied under the same degradation conditions. It should be noted that the absorption plateau for blank MO and pristine cashmere shows the stability of MO in its original solution, and that pristine cashmere fabric has no photocatalytic ability under UV light exposure. However, cashmere fabric with 10% and 5% TiO_2_ coating exhibited strong photocatalytic abilities towards MO with total degradation percentages of 96% and 92%, respectively, after 3 h of irradiation. Additionally, the corresponding color change of the MO solution is shown in Figure 4e, where the color of MO in contact with cashmere fabric with 5% and 10% TiO_2_ coatings completely disappeared after 3 h of irradiation, while the control samples had no color change. Figure 4d reveals that the photocatalytic degradation of MO fits pseudo-first-order kinetics, ln (C/C_0_) = k t, where C is the concentration of MO at time t (min), C_0_ is the initial concentration of MO solution, and the slope k is the reaction rate constant (min^−1^), which was 0.0138 min^−1^ and 0.0186 min^−1^ for 5% and 10% TiO_2_ nano-coating, respectively.

From the above, the TiO_2_ nano-coatings on cashmere, as a photocatalyst, exhibit a self-cleaning property in the degradation of MO in the presence of UV light. The basic mechanism of the process [30] is that, upon the absorption of light energy (hv ≥ 3.2 eV), photo-excited electron-hole pairs are generated within TiO_2_ where the electrons transfer from the valence band of TiO_2_ to its conduction band. Subsequently, the electrons diffuse to the surface of TiO_2_ to react with adsorbed O_2_, producing reactive oxygen radicals. On the other hand, the holes left in the valence band of TiO_2_ photo-oxidize H_2_O to form highly oxidizing hydroxyl radicals. Consequently, the generated radicals break down the surface-adsorbed organic contaminants into carbon dioxide and water.

**Figure 5:**
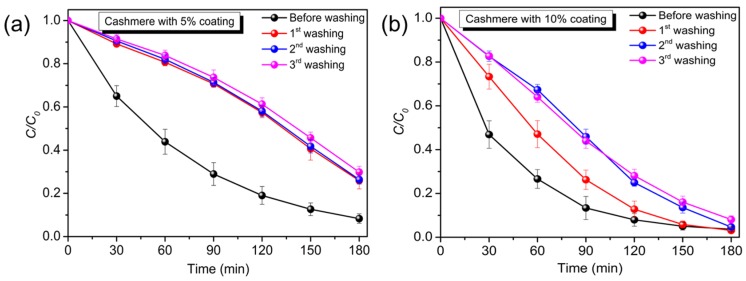
Photocatalytic activity of (**a**) 5% and (**b**) 10% TiO_2_-coated cashmere fabrics before and after washing (Error bar: three times).

Fabrics are subject to frequent washing, so the stability of the nano-coating on fabric is a vital requirement in view of its practical applications. As a delicate fiber, cashmere undergoes shrinkage and fiber damage when conventional detergent is used. Therefore, nonionic detergent was applied in the sequential washing testing to simulate the dry-cleaning process. The photocatalytic MO degradation performance was conducted after each wash cycle to study the stability of the coatings. The change of MO absorbance after each wash cycle is presented (Figures S3–S5). Figure 5 shows the photocatalytic activities of cashmere fabric after the 1st, 2nd, and 3rd wash cycles as compared with that before washing. It is noted that the photocatalytic properties of cashmere fabric with 5% TiO_2_ nano-coating (Figure 5a) exhibited some drop after the 1st wash, which could be explained by the loss of loose nanoparticles on or within the fabric structure. However, the activities remained unchanged in the subsequent wash cycles. On the other hand, compared to 5% TiO_2_-coated cashmere, 10% TiO_2_-coated cashmere fabric (Figure 5b) showed a lower loss of performance after the 1st wash, but a further drop was also observed after the 2nd wash. This may result from the loss of the thicker [14] and rather excessive coating on the fabric, as discussed in the surface morphology study (Figure 3c). Nevertheless, no significant drop was observed after the 2nd wash cycle. Besides, it is noted that the endpoint of each washing well overlapped, which further proves the photoactivity and suggests the stability of the TiO_2_ nano-coating is due to covalent bounding with the surface groups (–NH, –SH, –OH, –CH) [15] of cashmere fibers rather than physical deposition. The UV-vis diffuse reflectance spectra of the coated cashmere after each washing (Figure S6) further demonstrates that the loss of TiO_2_ has a negligible influence on the UV absorbance. These results further demonstrate that, without additional chemical or physical modification, cashmere can reach a good binding with nano-crystalline TiO_2_, which outperforms wool. The main difference between cashmere and wool is associated with the physical and morphological properties rather than chemical characteristics, where both have similar chemical structures with various amino acids [31]. While cashmere has predominantly mesocortical cells, wool possesses major paracortical cells resulting in a higher microfibril packing density and order in cashmere compared with wool [32]. Additionally, the peculiar fineness and scale structure makes cashmere superior to wool. These factors account for the difference in fiber functionalization. The one-step process also minimizes the impact on the intrinsic properties and hand-feel of cashmere. 

**Figure 6:**
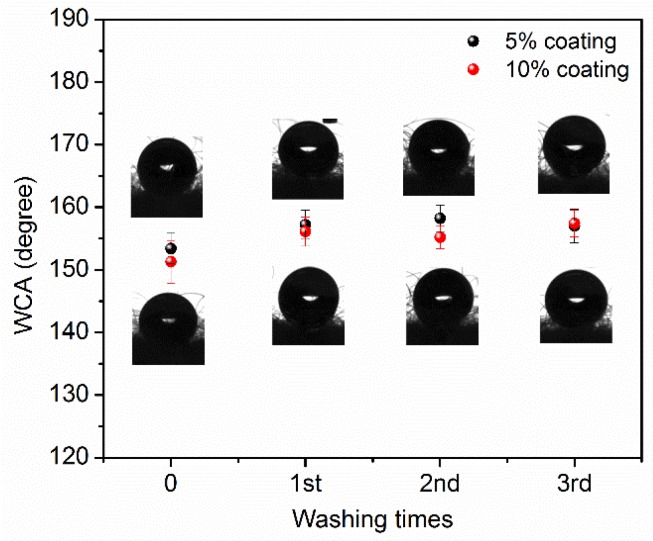
Water contact angle of 5% and 10% TiO_2_-coated cashmere before and after washing.

The wettability of a surface depends on the nature of the surface. In addition, a surface hydrophobicity increases with increasing surface roughness [33]. Due to the nature of cashmere, it possesses a hydrophobic surface property with a WCA of 150° ± 3° (Figure S7), which is ascribed to the inherent surface microstructure morphology of cashmere composed of air gaps between fibers. When a water droplet contacts with cashmere, the interface of the water droplet and the cashmere surface corresponds to the classic Cassie-Baxter model [1], where a liquid in the surface cavities entraps the air, maximizing the water and air interface area, resulting in the formation of spherical droplets with a higher roughness (lower hysteresis) and WCA. Figure 6 displays the WCA of TiO_2_-coated cashmere fabric before and after each washing. Before washing, the coated cashmere exhibited a slightly higher WCA than the pristine cashmere, resulting from the increase in surface area and roughness, which in turn increases the hydrophobicity. It is noted that both 5% and 10% TiO_2_-coated cashmere show the same trend of WCA, with a slight increase (from 153° ± 2° to 157° ± 2°) after first washing and remaining stable after sequential washing.

## 4. Conclusions

We have successfully developed a cashmere fabric with UV-induced photocatalytic self-cleaning properties by applying an anatase TiO_2_ colloid that is free of inorganic acids and organic solvents at low temperature via a one-step process. With the anatase nano-coating, the cashmere fabric exhibited significant photocatalytic properties towards the degradation of methyl orange, where the photocatalytic reaction was found to fit pseudo-first-order kinetics. In addition, the nano-coating on cashmere fabric exhibited good washing stability. After three wash cycles, coated cashmere fabric was able to retain its photocatalytic activities and WCA. Combining the photocatalytic activity with superhydrophobicity is a promising approach to develop effective self-cleaning surfaces.

## Supplementary Materials

The followings are available online at www.mdpi.com/1996-1944/10/12/1414/s1. Figure S1: EDS of 10% TiO_2_-coated cashmere. Figure S2: UV-vis diffuse reflectance spectra of pristine and coated cashmere. Figure S3: Absorption spectra of MO in presence of cashmere fabrics with (a) 5% and (b) 10% TiO_2_ nano-coatings after the 1st washing. Figure S4: Absorption spectra of MO in presence of cashmere fabric with (a) 5% and (b) 10% TiO_2_ nano-coating after the 2nd washing. Figure S5: Absorption spectra of MO in presence of cashmere fabric with (a) 5% and (b) 10% TiO_2_ nano-coating after the 3rd washing. Figure S6: UV-vis diffuse reflectance of cashmere with (a) 5% TiO_2_ coating and (b) 10% TiO_2_ coating after washing. Figure S7: Optical image showing water contact angle on pristine cashmere.
