# Supplementary material for: UV-Induced Photocatalytic Cashmere Fibers
Source: Materials (Basel). 2017 Dec 11;10(12):1414. doi: 10.3390/ma10121414 (PMC5744349; doi:10.3390/ma10121414)
Supplement: Supplementary file 1 [file materials-10-01414-s001.pdf]

materials

Supplementary Material

UV-Induced Photocatalytic Cashmere Fibers

Lingyun Wang and Walid A. Daoud*

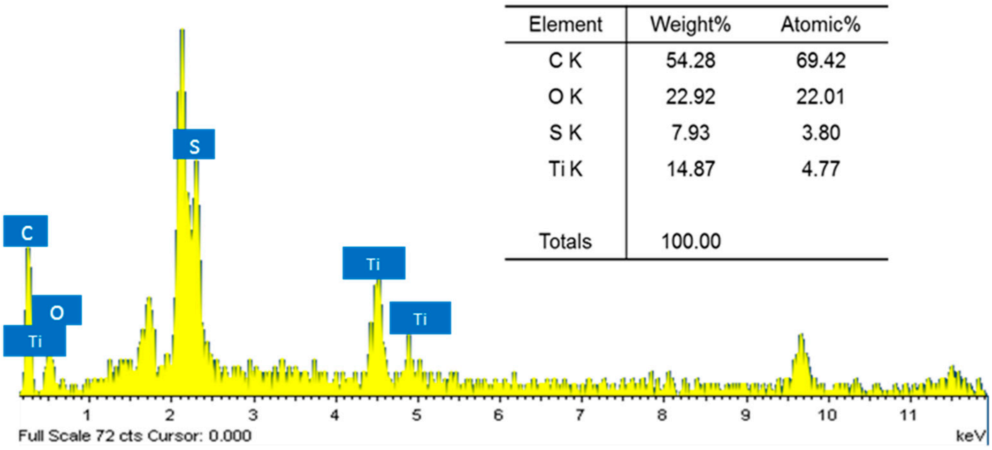

Element	Weight%	Atomic%
C K	54.28	69.42
O K	22.92	22.01
S K	7.93	3.80
Ti K	14.87	4.77
Totals	100.00	

Figure S1. EDS of 10% TiO_2-coated cashmere.

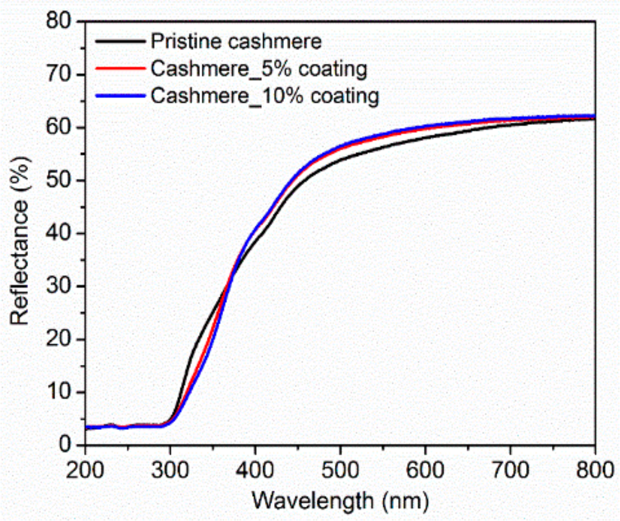

Figure S2. UV-vis diffuse reflectance spectra of pristine and coated cashmere.

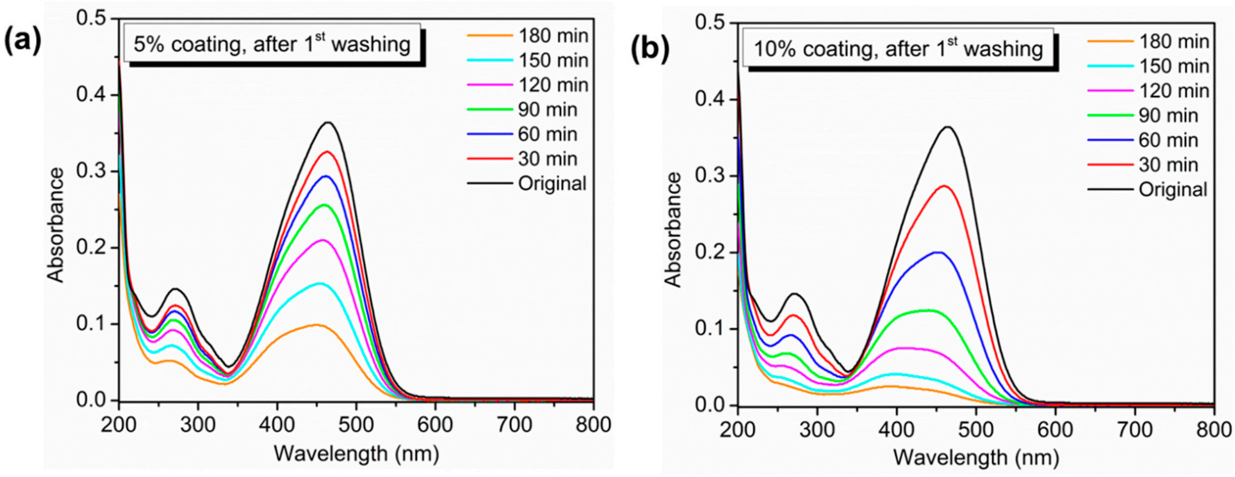

Figure S3. Absorption spectra of MO in presence of cashmere fabrics with (a) 5% and (b) 10% TiO_2 nano-coatings after the 1st washing.

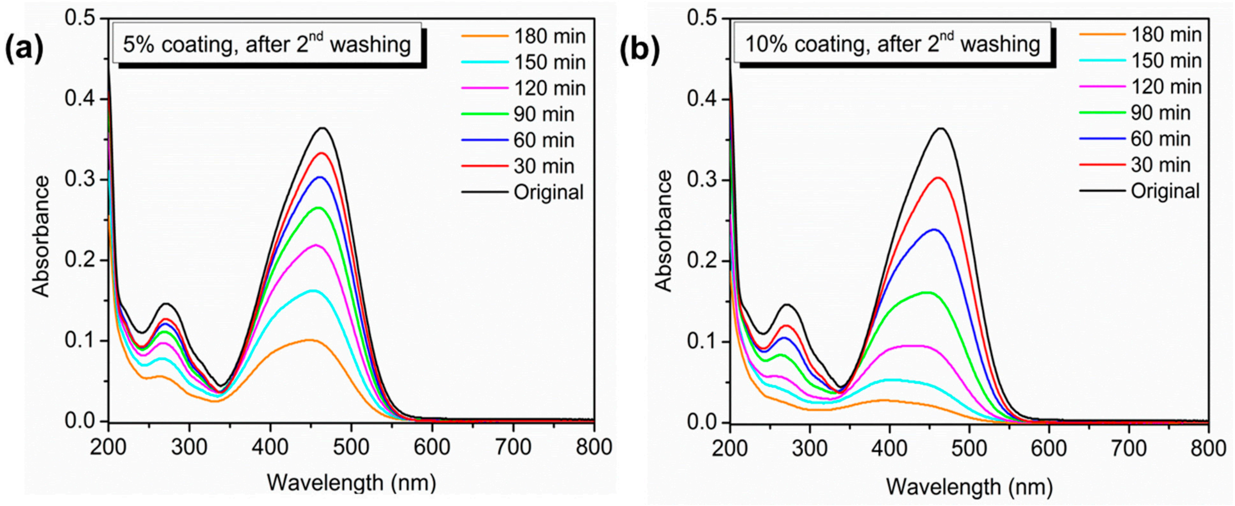

Figure S4. Absorption spectra of MO in presence of cashmere fabric with (a) 5% and (b) 10% TiO_2 nano-coating after the 2nd washing.

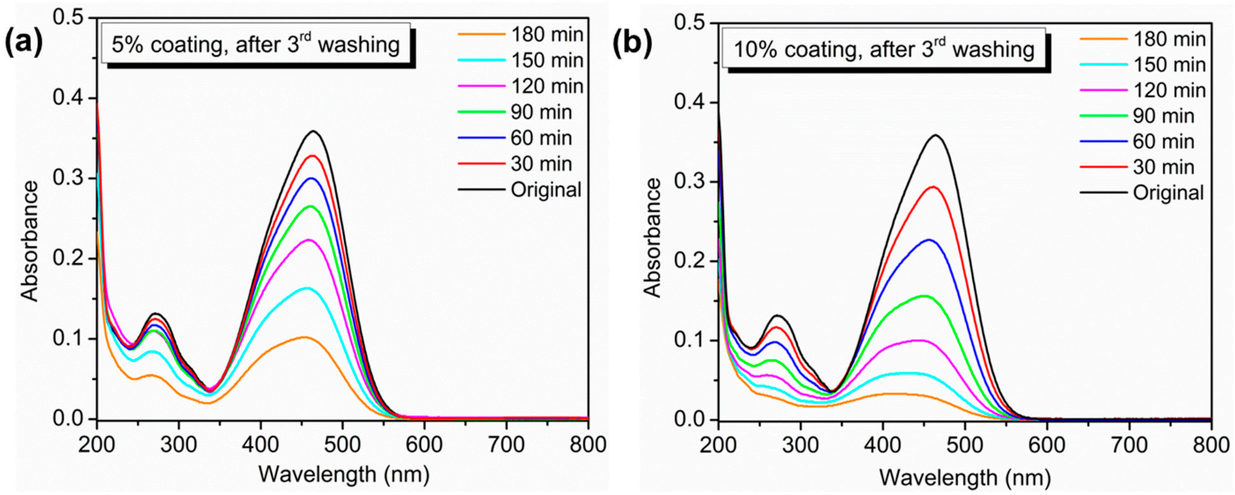

Figure S5. Absorption spectra of MO in presence of cashmere fabric with (a) 5% and (b) 10% TiO_2 nano-coating after the 3rd washing.

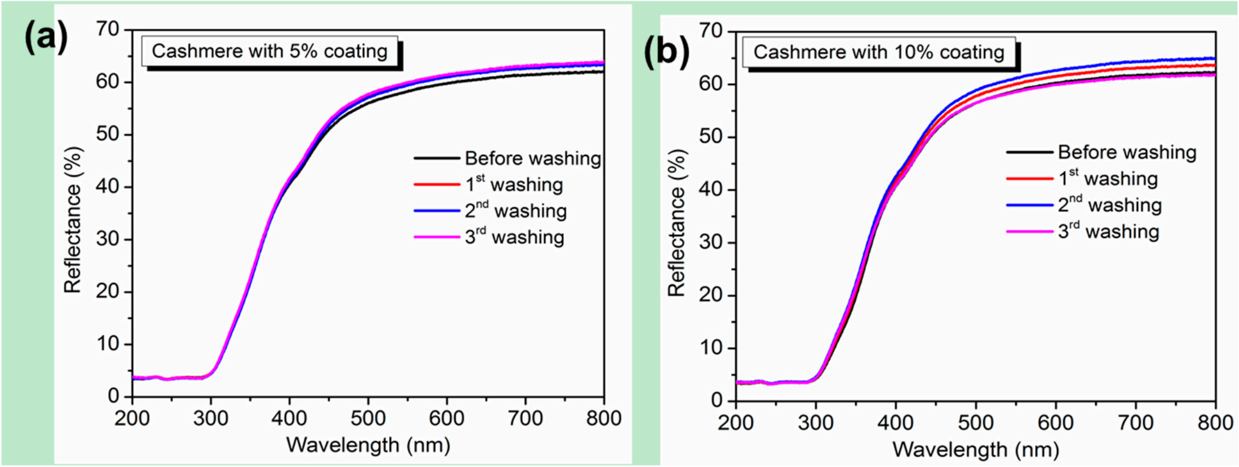

Figure S6. UV-vis diffuse reflectance of cashmere with (**a**) 5% TiO_2 coating and (**b**) 10% TiO_2 coating after washing.

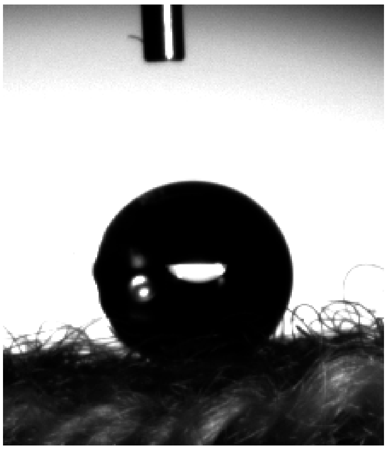

Figure S7. Optical image showing water contact angle on pristine cashmere.
